# Best evidence topic: Does the presence of a large gallstone carry a higher risk of gallbladder cancer?

**DOI:** 10.1016/j.amsu.2020.12.023

**Published:** 2020-12-28

**Authors:** Talal M. Alshahri, Sabry Abounozha

**Affiliations:** a Department of General Surgery, Imam Abdulrahman Alfaisal Hospital, Riyadh, Saudi Arabia; b Department of General Surgery, Northumbria Healthcare NHS Foundation Trust, Northumbria, UK

**Keywords:** Gallbladder cancer, Gallbladder adenocarcinoma, Large gallstone, Large gallbladder stone, Gallstone 3 cm

## Abstract

A best evidence topic has been constructed using a described protocol. The three-part question addressed was: In a patient with symptomatic gallstone disease does the presence of a large sized gallstone associated with a higher risk of gallbladder cancer?

Using the reported search, 3876 papers were found. 6 studies were deemed to be suitable to answer the question. The outcome assessed was the relationship between the presence of large sized gallstones and the risk of gallbladder cancer. It appears from the current available evidence that there is a strong association of a large sized gallstones and gallbladder cancer. Larger stones (>3 cm) have the greatest risk to develop gallbladder cancer, especially in symptomatic gallstone disease patients. Authors recommend special care for this patient's group and to warrant cholecystectomy when the clinical condition allows.

## Highlights

• A strong relationship between the gallstone size and gallbladder cancer.
• Stones larger than 3 cm have the highest risk.
• Cholecystectomy is warranted when the clinical condition allows.

## 1 Introduction

A Best Evidence Topic was constructed based on a structured protocol. This is described by the International Journal of Surgery [1].

## 2 Clinical scenario

You are a general surgery registrar, reviewing a patient with symptomatic gallstone disease in a general surgery outpatient clinic. You are discussing the ultrasound finding with him. The ultrasound scan shows the presence of a large gallstone which measures 3 cm. The patient asks you whether this finding is associated with higher risk of gallbladder cancer? What would you answer?

## 3 Three-part question

In [a patient with symptomatic gallstone disease] does [the presence of a large sized gallstone] associated with [a higher risk of gallbladder cancer]?

## 4 Search strategy

Medline® 1946 to November 2020 and Embase 1974 to 2020 using OVID interface:

[large gallstone OR large gallbladder stone OR size gallstone OR size gallbladder stone] AND [gallbladder cancer OR gallbladder adenocarcinoma]

Medline using the PubMed interface:

[large gallstone OR large gallbladder stone OR size gallstone OR size gallbladder stone] AND [gallbladder cancer OR gallbladder adenocarcinoma]

The results were limited to English articles and human studies.

## 5 Search outcome

**Fig. 1:**
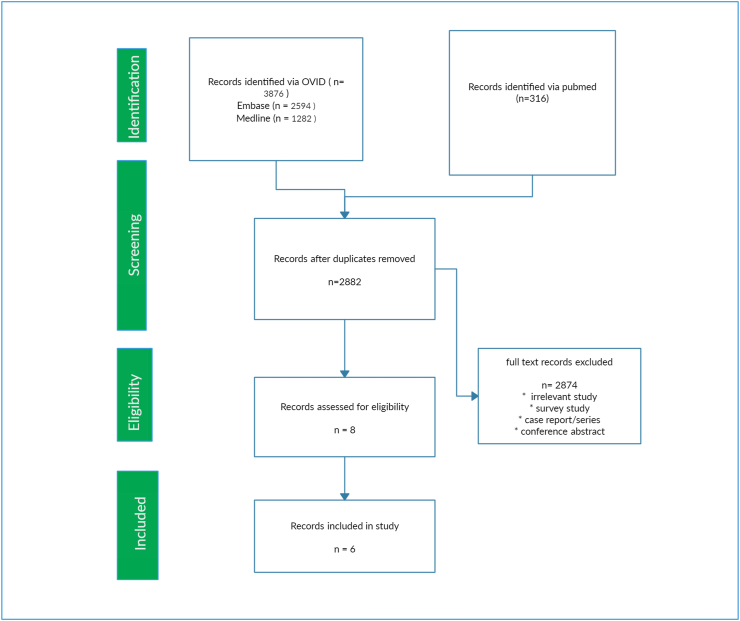
PRISMA flow chart.

A total of 3876 papers were found using OVID and 316 using the PubMed interface. 2882 papers were identified after duplicates were removed. Out of these, 2874 papers were excluded because they were irrelevant based on titles and abstracts. 8 full-text articles were screened and assessed for eligibility. From these, six papers were identified that provided the best evidence to answer the question. Eligible articles were defined as those articles that compared large gallbladder stone and risk of gallbladder cancer. The search strategy process is detailed in Fig. 1.

## 6 Result

(See Table 1).

**Table 1:** Results.

Author, date of publication, journal and country	Study type and level of evidence	Patient group	Outcomes	Key results	Additional comments
LOWENFELS et al. [2], 1989, International Journal of Epidemiology, USA	Retrospective cohort study, level III	The study included 1676 female patients. 25 of them identified to have gallbladder cancer. They used histopathology reports for patients whom surgery had been performed for cholelithiasis, and/or gallbladder cancer during the seven-year period 1979–1985	To investigate the relation between stone size and gallbladder cancer	They found a strong relationship between gallstone size and gallbladder cancer. Large stones (>3 cm) were found in 40% of patients with gallbladder cancer. The relative risk for gallbladder cancer in subjects with stones ≥3 cm was 9.2 compared with subjects with stones <1 cm. (95% confidence interval: 2.3–37)	Multicentre, large sample size, interracial, restricted to female patients and 3 races (white, black, native Americans), study was reported based on histopathology reports for symptomatic patients only
Csendes et al. [3], 2000, Journal of gastrointestinal Surgery, Chile	Prospective case control study, level IV	The study included 592 female patients divided into three groups: (A) 78 asymptomatic patients with gallstones; (B) 365 symptomatic patients with gallstones and (C) 149 patients with gallbladder carcinoma, during 1995–2000	To determine the number and the size of gallstones in patients with gallbladder carcinoma compared to asymptomatic and symptomatic female patients with gallstones	Patients with gallbladder carcinoma had significantly larger stones, regardless of the number of stones present (P < 0.001)	Single centre, large sample size, restricted to female patients
VITETTA et al. [4], 2000, ANZ Journal of Surgery, Australia	Retrospective Cohort Study, level III	Study consisted of 439 patients who underwent a cholecystectomy for gallstone disease. 14 patients (3.2%; 95%CI: 1.8–5.3) were diagnosed with gall bladder carcinoma, of which there were 11 women and three men, 1980–1994	Study reviewed the occurrence of gall bladder carcinoma in patients who underwent a cholecystectomy for gallstone disease	The author postulated that gall bladder carcinoma may be intimately associated with large or numerous cholesterol gallstones	Multicentre, large sample size, the study did not address the relation between acalculous gall bladder and gallbladder cancer
Diehl et al., 1983 [5], JAMA, USA	Case control study, level IV	The study included three controlled groups. 81 gallbladder cancer, 80 benign gallbladder controls, 66 non gallbladder controls. during 1976–1980	The purpose of this study was to determine risk factors that might interact with gallbladder stones to identify a high-risk subgroup for gallbladder cancer	Persons with large gallstones were found to be at increased risk for cancer. Stone diameter of 2.0–2.9 cm the odds ratio was 2.4; for stones >3 cm or larger the ratio was 10.1	Multicentre, the finding was not hypothesized before the study, small sample size, some selectively missing data which can raise potential bias, not sure that some of the patients in non-gallbladder controls have actually gallstone disease “as they can be asymptomatic”
Andrea et al. [6], 2015, Annals of surgery, Italy	Case Control study, level IV	The study Compared data from 2942 patients with benign biliary tract disease with those of 75 patients with gallbladder cancer, 20-year long study	The purpose of this study was to identify the high-risk group patients to develop gallbladder cancer which could benefit from preventive cholecystectomy	Patients with large gallstone of more than 3 cm at increased risk to develop gallbladder cancer. The increased risk odds ratio of developing gallbladder cancer has been reported to be 10.1 for gallstones larger than 3 cm versus stones smaller than 1 cm	Small sample size, a letter to editor has been found instead of a full article
Moerman et al. [7],1993, Scandinavian Journal of Gastroenterology, Netherlands	Case control study, level IV	43 cases and 98 controls with stones that could be classified by size were included in the analysis, cases were defined as patients with a surgical diagnosis gallbladder cancer while control defined as patients who underwent a cholecystectomy for a benign gallbladder disorder excluding carcinoma in situ, between 1983 and 1989	the relationship between stone size and gallbladder cancer	no association was found between the size of the largest stone within the gallbladder and gallbladder cancer in a matched hospital-based case-control study of surgical patients	Multicentre, small sample size, the patient group is mostly female and white ethnicity, Since a substantial number of patients with gallbladder cancer will not undergo surgery, the present findings cannot be generalized without caution to all patients with gallbladder cancer, relied on ultrasound for the size of gallstones

## 7 Discussion

Gallbladder cancer is a rare malignancy and as a result of its rarity, it is understudied [8]. It has been established that the presence of gallstones is an important risk factor [9]. Diehl et al. [5] conducted a case control study in 1983 to determine the risk factors for gallbladder cancer. Surprisingly, without a structured hypothesis before the study, they concluded a strong association between the presence of a large sized gallstone and gallbladder cancer. Moreover, they found that a stone diameter of 2.0–2.9 cm is associated with the odds ratio of 2.4 while for stones of 3 cm or larger the ratio was 10.1 for cancer.

In 1989, Lowenfels et al. [2] conducted a cohort retrospective study which included 1676 female patients. Twenty-five of them were identified to have gallbladder cancer. They used histopathology reports for patients whom surgery had been performed for cholelithiasis and/or gallbladder cancer. The authors found a strong relationship between gallstone size and gallbladder cancer. Large stones (>3 cm) were found in 40% of patients with gallbladder cancer. The relative risk for gallbladder cancer in subjects with stones ≥3 cm was 9.2 compared with subjects with stones <1 cm. (95% confidence interval: 2.3–37).

In 2000, Csendes et al. [3] conducted a prospective case control study which included 592 female patients divided into three groups: (A) 78 asymptomatic patients with gallstones; (B) 365 symptomatic patients with gallstones and (C) 149 patients with gallbladder carcinoma. They concluded that Patients with gallbladder carcinoma had significantly larger stones, regardless of the number of stones present (P < 0.001).

In the same year, Vitetta et al. [4] concluded that gall bladder carcinoma may be intimately associated with large or numerous cholesterol gallstones after the retrospective cohort study they conducted.

In 2015, Andrea et al. [6] conducted a case control study which Compared data from 2942 patients with benign biliary tract disease with those of 75 patients with gallbladder cancer. They concluded that patients with large gallstones of more than 3 cm at increased risk to develop gallbladder cancer. The increased risk odds ratio of developing gallbladder cancer has been reported to be 10.1 for gallstones larger than 3 cm versus stones smaller than 1 cm.

In contrast to all of the above-mentioned studies, Moerman et al. [7] conducted a case control study in 1993 which included 43 cases and 98 controls, cases were defined as patients with a surgical diagnosis of gallbladder cancer while control defined as patients who underwent a cholecystectomy for a benign gallbladder disorder excluding carcinoma in situ. They concluded that no association was found between the size of the largest stone within the gallbladder and gallbladder cancer.

## 8 Clinical bottom line

Five out of six studies have found a strong relationship between the large sized gallstones and gallbladder cancer. Larger stones (>3 cm) have the greatest risk to develop gallbladder cancer, especially in symptomatic gallstone disease patients. Authors recommend special care for this patient's group and to warrant cholecystectomy when the clinical condition allows.

## Ethical approval

Not applicable.

## Sources of funding

None.

## Author contribution

TA: conducted the literature search, structured PRISMA chart and interpretated the results.

SA: conducted the literature search, interpretation of the results and wrote the paper.

## Registration of research studies

1. Name of the registry: Not applicable.

2. Unique Identifying number or registration ID:

3. Hyperlink to your specific registration (must be publicly accessible and will be checked):

## Guarantor

Talal M Alshahri (TA) and Sabry Abounozha (SA)

## Consent

Not required.

## Declaration of competing interest

None.
